# Professional Silos or Professional Integration? Exploring the role of the basic science disciplines in healthcare professionals’ professional identities

**DOI:** 10.15694/mep.2018.0000241.1

**Published:** 2018-10-25

**Authors:** Georgina Willetts, Michelle Lazarus

**Affiliations:** 1 Swinburne University; 2 Monash University

**Keywords:** Professional Identity, Basic Science education, Skill development

## Abstract

This article was migrated. The article was marked as recommended.

Professional identity (PI) is an important topic within modern healthcare curricula; presently, explorations of this topic focus on the impact of clinical experience. We sought to explore the impact of science education on PI from healthcare practitioners’ perspectives using a qualitative approach. While this work is still in relative infancy, we found an unexpected outcome - that healthcare workers perceive their science knowledge as a central component of their PI. This work begins to unpack the complicated and previously poorly explored interplay between the sciences and clinical contexts have on practitioners’ PI. Progression of this research area may help improve integration and explicit linking of healthcare sciences with both clinical education and PI development.

## Introduction

Preparing healthcare students for their future requires a multifaceted learning environment comprised of: knowledge acquisition, skill competency, and development of a professional identity ( Jones, Higgs et al. 2001). There is increasing interest in exploring whether skill development and professional identity (PI) formation are impacted through both science education *and* clinical placements (Monrouxe 2010).

The current trend in healthcare education is toward promotion of early clinical experiences, frequently at the expense of basic science teaching ( Drake, McBride et al. 2009). We identify some of the current challenges between the (perceived) dichotomy of basic science and clinical experience. Some healthcare educational programs, such as nursing, tend towards more holistic and inclusive pedagogical approaches ( Lutzen, Johansson et al. 2000). This holistic view of the healthcare environment drives nursing curricula towards favouring early and comprehensive integration of clinical skills with the science paradigms underpinning these skills; thus nurses may not view the sciences as a standalone competency but as an integrated part of their PI.

In contrast, the Flexner model typical of 20 ^th^ century medical schools separates pre-clinical coursework with the basic sciences preceding clinical coursework ( Flexner, Pritchet et al. 1910), providing students with focused, in-depth and discrete science education. The tension between the sciences and the clinic is, thus, most striking in medical education ( Bandiera, Boucher et al. 2013). Current practicing medical doctors (MDs) and bachelor of medicine, bachelor of surgery (MBBS, BMBS) graduates (and participants in this study) predominately went through a Flexner-type medical school curriculum.

## Method

The aim of this project was to *scope initial perceptions of the role of basic science on healthcare professionals PI* with an intent to inform the development of future research questions/projects exploring this phenomenon. We sought to investigate these questions by engaging a group of international clinical professionals attending an international medical skills conference in 2016. Participants attended the conference workshop knowing the research intent (Monash University human ethics board approval ID 7773).

The workshop utilised a focus group method which was audiotaped (initial themes identified with participant participation) and then transcribed. Thematic analysis was undertaken and the results are briefly presented in this paper. This was a unique methodological approach underpinned by constructivism. The interpretation and meanings of these diverse healthcare professional roles was explored. The four key questions posed at the workshop were:

• **What (if any) clinical contexts translate into basic science teaching?**
• **What is the skillset for science vs. clinical teachers?**
• **What are learning outcomes expected in science vs. clinical courses?**
• **Does science teaching have any role or influence on healthcare students’ PI?**

Validity and rigour of analysis was achieved through processes of cross-checking and cross validating against the data and the researchers’ interpretation of the data ( Hanson, Balmer et al. 2011). This was further validated through reflexivity undertaken throughout the analysis phase, seeking not to eliminate researcher expertise but rather support the analysis (Ramani and Mann 2016). The involvement of both a healthcare professional and a basic scientist at all stages of the research further ensured rigour through their collective and yet diverse backgrounds.

Participant (n=17) demographics included nine doctors, four nurses, one physiotherapist and three academic scientists. There were 11 self-identified females and 6 males. Sixteen of the participants were over the age of 40.

## Results

Three themes, contextualized below, were identified after dual coding, through constant comparative method, and analysis: 1. The perceived dichotomy between Clinical and Basic Science, 2. Teaching in silos 3. PI and the clinical context ( Table 1).

**Table 1:** 

Participant Demographics (n=17)	Discipline Medicine (n=9) Nursing (n=4) Physiotherapy (n=1) Academic scientist (n=3)	Gender Females (n=11) Males (n=6)	Age 40+ (n=16) 30-34 (n=1)
Themes	Perceived dichotomy between Clinical and Basic Science	Teaching in silos	Professional Identity and the clinical context
**Theme Definition**	The divide between the clinical areas and the basic science areas within healthcare education.	Teaching limited to one area/specialty without recognition of the potential for interrelated learning.	The development of discipline specific, behaviours values and ideals, expressly related to healthcare practice contexts.
**Quotes**	*I feel as though basic scientists think about problems differently.*	*Have [you] ever looked at a curriculum mapping, it will show clinical skills, and it’s a separate thing from the basic science courses, and I want that to stop.*	*I think the knowledge that we draw upon depends on our professional identity*
	*So it’s very difficult to get them on hand ... let’s say, biochemist is they have never seen a patient and they have no idea what is relevant and they have spooky ideas sometimes about what’s going on in the human body; so you see, it is a problem.*	*I think it goes back to the point that if you’re learning it not linked to patients. It doesn’t make sense.*	*that’s what we’re finding actually, in the basic science classroom, is that these covert values are influencing more of the professional identity than any of the knowledge that we’re providing.*
	*the various departments, anatomy being one of them, are heavily politicised in terms of their slice of the pie, and you don’t want to share the teaching because when you share the teaching, they share the budget*		*the difference between physician or an allied health provider, I’d say is that they are deeper, like have a deeper basic science.... it’s the deep sciences that separates the identity of a physician..... not so much the deep knowledge, but the application of that knowledge*

## Discussion

The discourse between basic and clinical science in medical education is reinforced and historically embedded in curricula ( Finnerty, Chauvin et al. 2010). We identified that despite a desire to integrate, hurdles and challenges with funding, timetabling, and basic infrastructure existed. There was clear underlying perception that scientists had a lack of working knowledge of clinical contexts from which to draw upon within medical education, decreasing their credibility; the medical doctors felt they themselves were best placed to teach medical students the sciences. The time/cost was often prohibitive for medical doctors to contribute to teaching, particularly in the preclinical years. As a result, there was a begrudging reliance on scientist educators as this was considered a compromise on education quality. *Interestingly other health professional participants did not reflect this*; in contrast they valued the contribution of scientists’ depth of knowledge.

Workshop participants agreed that true integration between science and clinical skills was ultimately the goal. Science teaching early in curricular progression was not unique to medicine; all health professions require foundational science knowledge upon which to build. There was a difference between temporal integration (i.e. timetabling) and pedagogical integration (i.e. content integration in a single learning experience). Non-medical health profession courses appeared to engage the latter integrated approach more often. Medical education struggled with pedagogical integration; to Vertical integration was a proposed solution ( Finnerty, Chauvin et al. 2010). There were difficulties operationalizing this mostly because of funding/infrastructural opposition. Ultimately, *it was identified that all health professions need science but for each of the professions the meanings and application are different.*

The potential influence of science on PI was a concept not previously considered, however, medical doctors saw science education as “context free”. Physiotherapy and Nursing, alternatively, made the link between sciences and clinical contexts by incorporating professional behaviours within laboratory work and consistently linking back to PI. Interestingly, for medicine the role of science in PI development was initially mostly unrecognized; when considered by participants *science was identified as profoundly implicit in a medical doctor’s PI.*

## Implications and Conclusions

Basic sciences, and their role in healthcare education, are fraught with debate ( Koens, Custers et al. 2006). Questions remain on how to teach it, when to teach it, who should be teaching it and how much should be taught ( Pawlina 2009). Core in these discussions, particularly in medical education contexts, is that sciences are limited to soley knowledge acquisition ( Koens, Custers et al. 2006). We questioned this perception within a group of diverse healthcare practitioners; what resulted was a passionate debate about the role of sciences (and scientists) in healthcare education; *the fervent nature of the debate itself suggests that science is not separate from healthcare PI, rather science are integral.* *Our findings suggest moving away from seeing “the sciences” and the “the clinical” as separate entities, but rather as a continuum within professional role and identity development.* Our results suggest a need for deeper understanding and exploration focused on identifying the impacts of science teaching on PI as this may help healthcare students (and practitioners) improve pedagogical integration of sciences and PI.

## Take Home Messages

The importance and potential influence of Basic Sciences to the development of Professional Identity across the health professions has afforded limited research. This is an important area requiring further investigation and opportunity to improve both peadagogy and practice.

## Notes On Contributors

**Associate Professor Georgina Willetts RN RM DEd** is Head of Discipline Nursing Swinburne University of Technology. She has extensive experience in Healthcare practice, management and education. Focusing on curriculum development, design, implementation and evaluation.

**Michelle D. Lazarus** **PhD** is a senior lecturer and Monash Education Academy Fellow whose primary teaching role is in the medical anatomy courses. Her work focuses on exploring the role of basic sciences on medical students’ professional identity development. She also develops and runs workshops facilitating basic scientists’ transition into education research and scholarship.
