# Supplementary material for: Professional Silos or Professional Integration? Exploring the role of the basic science disciplines in healthcare professionals’ professional identities
Source: MedEdPublish (2016). 2018 Oct 25;7:241. [Version 1] doi: 10.15694/mep.2018.0000241.1 (PMC10711965; doi:10.15694/mep.2018.0000241.1)
Supplement: Peer review history

# Reviewer report: Reviewer response for version 1

**DOI:** 10.21956/mep.19710.r29488

**Published:** 2019-08-08

**Authors:** Richard Hays

**Affiliations:** 1 James Cook University

This review has been migrated. The reviewer awarded 3 stars out of 5 While this paper addresses an important topic in medical education, it is really more of an pilot qualitative exploration than a definitive study, aiming to identify issues relevant to the topic. Perhaps this should be stated more strongly, rather than thinking of it as a case study, which implies multi-perspective, multi-method analyses that produce lessons that may be relevant in similar contexts ( see Cheek et al. Improving case study research in medical education: Systematised review. Med Educ, 2017; 51: 1-8 Available: https://doi.org/10.1111/medu.13469). Softening of the findings may also be useful, as this may be the beginning of a series of conversations rather than an end. One possible omission stands out. I have found that horizontal integration in the early years (cellular, molecular and organ sciences teaching together) has been more of a problem that vertical integration (science to clinical). Have the authors any comments on that aspect?

Reviewer Expertise:

NA

No decision status is available

# Reviewer report: Reviewer response for version 1

**DOI:** 10.21956/mep.19710.r29490

**Published:** 2019-06-04

**Authors:** Ken Masters

**Affiliations:** 1 Sultan Qaboos University

This review has been migrated. The reviewer awarded 2 stars out of 5 An interesting case study exploring the role of the basic science disciplines in healthcare professionals’ professional identities. While the paper is interesting, there are limitations, and I feel that the authors are trying to address too much with the small number of participants. On the one hand, the classification of the paper as a “Case Study” means that the authors do not have to apply the same standards of sample sizing and selection that would be expected from a research paper; on the other, it means that the authors cannot draw inferences and conclusions affecting any wider group that those being studied.At the heart, the paper appears to be treading the line between, and attempting to conflate, two questions: (1) do the basic scientists influence the PI, (2) do the (medical?) clinicians perceive that the basic scientists influence the PI? These are two different questions, and need to be answered by two different groups of people. If one is addressing the first question, then there are only three people in the sample who can answer; if the second question, then there are nine. If one wishes to introduce the same questions for nursing and physiotherapy, this can also be done, but in those fields, the sample has only four representatives and one representative, respectively. These numbers are not enough to arrive at any real and useful conclusion beyond this narrow group. The number of quotations supplied in support of the themes is also extremely limited, and so reinforces the very narrow applicability of the results.As a result, statements like “Our findings suggest moving away from seeing "the sciences" and the "the clinical" as separate entities, but rather as a continuum within professional role and identity development” are not supported by the study. The most that can be said is that the study shows information from this small, non-representative group. While the topic is worth exploring further, at this stage, this is the limit of the findings, and to suggest changes based upon this small case study is inappropriate.So, a useful Case Study on a small group, but the inferences and conclusions are far too broad.

Reviewer Expertise:

NA

No decision status is available

# Reviewer report: Reviewer response for version 1

**DOI:** 10.21956/mep.19710.r29489

**Published:** 2018-11-07

**Authors:** Simonetta Ausoni

**Affiliations:** 1 Department of Biomedical Sciences- University of Padova- School of Medicine

This review has been migrated. The reviewer awarded 4 stars out of 5 As a scientists and medical educator, I really enjoyed reading this paper. The final message that basic sciences are perceived as essential knowledge to acquire health professional identity is interesting and should be investigated further. Developing strategies for teaching basic sciences in the medical courses is still a problem. Separation and poor integration of preclinical and clinical courses persist in many medical schools. Different solutions have been proposed over decades, but the enormous explosion of scientific discoveries requires continuous reshaping of the teaching contents and methodologies. If basic sciences play a major role in the development of professional identity, then my take home message from this study is that scientists and clinicians should promote more collaboration, not only for the purpose of scientific research, as they do all the time, but also for the benefit of teaching. It is true that scientists lack knowledge of clinical contents and clinicians lack deep scientific knowledge, but I’m afraid……I don’t see such a big effort to fill this gap in the setting of teaching. To conclude, I recommend reading this paper and hope the authors will extend this research. I have only a minor concern about the manuscript: the Results are presented in a too concise form. Of the three themes identified for analysis, only a limited number of quotes are reported and it is not even clear who said that (doctors, nurses… scientists?). More info about the professional activity of the study group members would be also helpful: for example, it is not clear to what extent each participant applies basic science knowledge in their professional activity. Are they involved in clinical research? Are they employed in hospitals? Fixing these points could improve quality of the manuscript.

Reviewer Expertise:

NA

No decision status is available

# Reviewer report: Reviewer response for version 1

**DOI:** 10.21956/mep.19710.r29487

**Published:** 2018-10-26

**Authors:** Felix Silwimba

**Affiliations:** 1 University of Lusaka

This review has been migrated. The reviewer awarded 5 stars out of 5 This is a very important study and report to discuss medical /health professional education. It applies to all regions of the world, super rich, rich, middle- or low-income economies. It underscores the need for contextualised training. The report has provided a strong link to the past and the evolution of modern medical education. that is the Flexner report of 1910. This report was done slightly over a 100 years ago. I suppose basic science education was not wide spread as it is today in the formal education system. Therefore, my worry is how does such a report remain relevant to medical education today, considering the explosion that has taken place in basic science education and technological development. Agree with the researcher take home message of need for research in basic sciences influence on professional identity. I suppose basic science education should be strengthened in the general education system and medical education should concentrate on the application of basic science knowledge, professional identity and health professional ethics. middle and low income countries should take the lead in this critical review.

Reviewer Expertise:

NA

No decision status is available
